# Wandering as returning? Rethinking family dynamics of Chinese gay men under neo-familism through transnational mobility

**DOI:** 10.3389/fpsyg.2025.1510499

**Published:** 2025-05-13

**Authors:** Haohua Wang

**Affiliations:** School of Sociology and Anthropology, Xiamen University, Xiamen, China

**Keywords:** Chinese gay men, transnational mobility, family dynamics, neo-familism, reconciliation

## Abstract

While extensive research has explored the causes of transnational mobility among Chinese gay men, there has been insufficient focus on the post-mobility challenges on how Chinese gay men reconcile with their family of origin. This study, grounded in Yan’s neo-familism framework, utilizes in-depth interviews with 20 Chinese gay men who have lived in the West for at least 1 year. It examines how transnational mobility enables these men to navigate their sexual identities in relation to their non-mobile parents. The findings indicate that transnational mobility alleviates family conflicts and facilitates the integration of gay identities into family structures through distinct reconciliation pathways fostering harmony and mutual understanding. This study significantly contributes to the field of sexual minorities in non-Western cultural contexts, particularly by illuminating the sophisticated family dynamics and reconciliation strategies that emerge in response to transnational mobility.

## 1 Introduction

In traditional Confucian Chinese culture, family values emphasize marriage and procreation, which creates considerable pressure on Chinese gay men to confront family traditions (Luo, 2022). The fear of heterosexual normative pressure and societal stigma often prevents many from openly acknowledging their sexual orientation (Liu et al., 2011). These pressures not only shape individual identity but also complicate the dynamics between gay men and their families, as they navigate societal expectations in an increasingly globalized world. As a response to these pressures, Some Chinese gay men perceive transnational mobility as a solution to the tension between family expectations and gay identity and as a means to evade judgment from society (Ponce and Chen, 2023). This desire for autonomy and self-expression is rooted in transnationalism, a concept referring to the sustained and multi-directional exchanges individuals maintain across national borders, which influence their social, economic, cultural, and political interactions (Schiller et al., 1995). By engaging in these cross-border exchanges, Chinese gay men are able to create spaces where personal identity and family obligations can be redefined, providing them with a degree of freedom that is often constrained within traditional societal norms. Influenced by the process of globalization and individualization (Altman, 1996; Roseneil, 2007), members of queer communities grappling with family tensions often leave their hometowns in pursuit of self-discovery and personal values. Many Chinese gay men may more likely choose to migrate to countries in the Global North, seeking not only economic opportunities but also greater freedom and societal acceptance (Choi, 2022). These countries offer stronger legal protections for LGBTQ+ individuals, including anti-discrimination laws, same-sex marriage legalization, and a more inclusive social environment. As Choi (2022) argues, such migration is driven by the desire to escape state oppression and family pressures while leveraging class-based capital to access opportunities for self-expression and life fulfilment.

This study aims to explore the process of which transnational mobility among Chinese gay men affects family dynamics with their non-mobile parents. Neo-familism refers to a resurgence of family centrality in modern contexts, blending traditional Confucian family values with contemporary individualistic aspirations (Yan, 2018). This hybrid framework emphasizes the family’s continued influence on personal decision-making processes, even as individuals seek autonomy in other areas of their lives. In the context of Chinese gay men, neo-familism shapes the tension between personal identity construction and fulfilling family expectations, creating a unique landscape of identity negotiation that is both modern and deeply rooted in tradition. This framework is particularly salient in the context of transnational mobility, where Chinese gay men, through migration, not only negotiate their sexual identities but also actively reshape family dynamics. Transnational mobility emerges as a transformative process that enables these men to reconfigure their positions within the family, fostering a dynamic interplay between personal identity formation and family reconciliation. The findings of this study demonstrate that mobility empowers Chinese gay men to reconcile these dual imperatives, creating spaces for dialogue and mutual understanding across generational divides. Concurrently with the promotion of intergenerational dynamics and the reinforcement of personal gay identities, a new transformation takes place within the family. This perspective also critiques aspects of queer identity studies that challenge the institution of the family, particularly those that view the family as a site of queer resistance (Edelman, 2020; Hordge-Freeman and Freeman, 2010).

Drawing upon life story interview data, this study elucidates the intricate mechanisms driving transnational mobility, its consequential impacts on family dynamics, and the pursuit of family acceptance by Chinese gay men in alignment with their identities. This study synthesizes prior research concerning the underlying tensions between anti-family sentiments and the dynamics of intergenerational relations within gay families. Employing transnational mobility as a comprehensive empowerment process sheds light on the potential evolution of intergenerational family dynamics among Chinese gay men. This research is structured around three key questions that align with the stages of the transnational mobility process: (1) How do early family dynamics between Chinese gay men and their non-mobile parents influence the decision of transnational mobility? (2) In what ways does transnational mobility act as a mechanism for empowerment and negotiation in the context of evolving family dynamics? (3) What pathways do Chinese gay men employ to navigate and reconcile family expectations with their gay identities? By integrating these questions, the study explores the intersections of migration, identity, and family, offering insights into the ways in which transnational mobility impacts the lives of Chinese gay men and their family dynamics.

## 2 Literature review

### 2.1 Queer migration, family dynamics and Chinese gay men’s transnational mobility

The contemporary landscape of queer migration and transnational mobility is characterized by a complex interplay of socio-political and cultural factors that significantly impact the experiences of LGBTQ+ individuals. Research indicates that queer migration is often motivated by the pursuit of safety, social acceptance, and opportunities for identity affirmation, particularly in regions where legal protections and societal acceptance remain limited (Fresnoza-Flot, 2021; Yang, 2024). The experiences of queer migrants are further complicated by intersecting identities related to race, class, and gender, shaping their migration pathways and outcomes (Ayoub and Bauman, 2019; Wimark, 2021; Zhou, 2021; Loa and Choi, 2024). For example, LGBTQ+ migrants frequently navigate precarious legal statuses and economic uncertainties that affect their integration into host societies, illustrating the multifaceted nature of their mobility experiences (Datta, 2022; Loa and Choi, 2024).

The relationship between queer migration and family dynamics is particularly crucial. Families can act as both sources of support and sites of conflict, affecting queer individuals’ migration trajectories. Many queer migrants navigate the tension between family loyalty and personal autonomy, as they confront expectations rooted in traditional family structures (Kong, 2023; Ponce and Chen, 2023; Boyd and Wei, 2024). This complexity is highlighted by research that illustrates how queer individuals leverage family ties to facilitate their mobility, striving for acceptance while managing the expectations of their families (Fresnoza-Flot, 2021; Sert and Turkmen, 2022; Choi, 2022; Vuckovic Juros, 2022).

While the discourse surrounding queer migration has expanded, there remains an under-exploration of non-Western contexts in existing literature. Much of the focus has been on Western experiences, which may not fully encapsulate the unique challenges and cultural dynamics faced by queer individuals in Asia, Africa, and Latin America (Ritholtz and Buxton, 2021; Chen, 2023; Novitskaya, 2023; Zheng, 2023; Held, 2023). Researchers have emphasized the importance of considering how family and cultural expectations uniquely influence the migration experiences of queer individuals in these regions (Zheng, 2023). For instance, studies indicate that queer migrants from non-Western backgrounds often encounter compounded pressures from family obligations and societal norms, which can significantly shape their mobility decisions (Camminga and Marnell, 2022; Held, 2023).

Focusing on Chinese gay men, the experiences of their transnational mobility reveal unique challenges and opportunities. Chinese gay men often negotiate family obligations against the backdrop of evolving queer identities, with pressures to conform to heterosexual norms, such as marriage and parenthood, acting as significant catalysts for their mobility (Choi and Luo, 2016; Liu, 2019; Tang et al., 2020). Existing studies have explored the challenges faced by Chinese gay men within the family and societal context. Luo (2022) examines the intricate ways in which *Jia* (family/home) influences migration motivations among young Chinese gay men, offering a temporal perspective that enriches the understanding of family impact on migration decisions. As Yang (2024) highlights, this demographic increasingly seeks to balance parental expectations with their sexual autonomy, fostering a detailed understanding of family roles. The concept of “yes, but not yet,” as articulated by Luo et al. (2024), encapsulates this ambivalence, suggesting that while Chinese gay men may defer family responsibilities, they maintain optimism about potential reconciliations.

Thus, this study aims to explore how transnational mobility functions not only as an escape from family pressures but also as a mechanism for negotiating and reconfiguring family relations within the context of evolving sexual identities. By situating this discussion within the framework of neo-familism, this research contributes to a more significant understanding of how mobility intersects with family dynamics in a transnational setting, emphasizing the transformative potential of queer migration experiences (Zheng, 2023).

### 2.2 The “yes, but not yet” status: post-mobility challenges

While family is often perceived as a primary catalyst for the mobility of gay men (Wimark, 2016), there is limited exploration of how the gay community navigates family dynamics during and after migration. Luo et al. (2024) introduce the concept of the “yes, but not yet” status, characterizing it as a transitional phase in intergenerational family relations among Chinese gay men. This status encapsulates an ambivalent space where Chinese gay men may defer family responsibilities while holding an optimistic outlook towards fulfilling these duties. Although they acknowledge the significance of family within societal and cultural contexts, many feel ill-equipped to meet the expectations imposed by both their families and a heteronormative society (Wong et al., 2019; Xie, 2023).

The “yes, but not yet” status offers a discriminating lens through which to understand the complexities of post-mobility challenges faced by Chinese gay men. It underscores the ongoing tension between family obligations and personal identity, suggesting that this status does not signify a definitive resolution to conflicts within family structures (Gong and Liu, 2022). Instead, it reflects a dual transformation process marked by both transnational mobility and life progression (Russell, 2018). This dynamic illustrates how Chinese gay men navigate the intricacies of their identities while aspiring to transcend the limitations imposed by traditional family roles.

Transnational mobility can function as a vital mechanism for negotiating these tensions, offering opportunities for individualized growth and reconciliation between Chinese gay men and their families. As Luo et al. (2024) argue, mobility should not merely be viewed as an escape strategy; it serves as a catalyst that facilitates dialogue and understanding between generations. Supporting this notion, Binnie and Klesse (2013) highlight the role of mobility in reshaping family dynamics, while Davies et al. (2018) from emphasizes to emphasize how such experiences provide a broader context for understanding personal identity and family dynamics.

However, these resolutions are often multifaceted, open-ended, and elusive, necessitating innovative theoretical frameworks to unpack the intersections of gay identities, intergenerational relations, and transnational mobility. The evolving context of neo-familism adds another layer of complexity, as individuals strive to balance family loyalty with their quest for personal authenticity (Kong, 2023). This ongoing negotiation encapsulates the “yes, but not yet” status, underscoring the significance of transnational experiences in shaping the identities and family dynamics of Chinese gay men.

### 2.3 Neo-familism: a framework for understanding reconciliation

An increasing number of scholars highlight the significance of gay identity development in relation to intergenerational family dynamics, aligning their insights with the principles of neo-familism (Quach et al., 2013; Kong, 2020; Shi et al., 2020). Emerging within the context of risk society and rising individualism, neo-familism elucidates the evolving landscape of family structures and dynamics. According to Yan (2021), family life encompasses material, spiritual, emotional, and existential dimensions, transitioning from a focus on ancestors to descendants. This framework offers a comprehensive understanding of parent–child relationship models prevalent in modernizing Chinese families (Yan, 2021). Neo-familism has profound implications for gay identity development, as it compels individuals to reconcile conflicting priorities: the desire for self-expression and the obligation to uphold family expectations. In the context of transnational mobility, Luo (2022) reveals how family obligations often motivate young gay men to migrate, using physical distance to create emotional space for identity exploration. Cai (2023) demonstrates that this negotiation extends to social practices, such as marriages of convenience, which allow Chinese gay men to navigate family pressure without completely severing ties. This intersection of identity, individualism, and family highlights the layered role neo-familism plays in shaping the experiences of Chinese gay men.

Neo-familism provides a valuable lens through which to examine family dynamics and individualization within the Chinese gay community. Neo-familism retains essential attributes of traditional familism, such as the family’s vital role in shaping daily life and prioritizing intergenerational bonds, alongside the moral significance of filial piety. The reinterpretation of filial piety reflects contemporary individual circumstances, creating tensions between personal desires and family expectations (Yan, 2010; Choi and Peng, 2016). Recent contributions from Chinese sociologists have shed light on the interactions between family dynamics and queer identities under this framework. For example, Wei and Yan (2021) discuss positive shifts in family acceptance of queer identities within the neo-familism paradigm, influenced by factors such as gender diversity and family interactions. Conversely, literature also documents strained dynamics that result in disruptions in family meaning-making, feelings of isolation, challenges during the coming-out process, and renegotiation of sexual orientation and reproductive responsibilities (Oswald, 2002; Kong, 2020). While some challenges mirror Western experiences, the patriarchal structure, reproductive obligations, and ethical norms entrenched in traditional family systems continue to impact Chinese gay men, eliciting feelings of anxiety and ambivalence.

Nevertheless, attributes of neo-familism have garnered attention. Some queer sociologists propose that mobility serves as a generalized strategy to alleviate intergenerational tensions, allowing individuals to distance themselves from family conflicts (Weintrob et al., 2021). Employing the neo-familism framework reveals that the reconciliation of gay identities with intergenerational relations extends beyond mere disengagement or the “not yet” status, leading toward a more profound understanding of the complexities involved.

This study underscores the adjustment processes by which Chinese gay men maintain family ties despite conflicts arising from the divergence between their evolving gay identities and family expectations. Scholars have traditionally framed queer mobility in terms of how “family makes or limits you,” highlighting narratives of powerlessness and distress in mobility journeys. I propose reframing the question to “What kind of family do I want?” This perspective empowers mobility from a queer standpoint, exploring return within the discourse of identity rather than solely in physical terms (Luo, 2022; Luo et al., 2024).

## 3 Research methods

The study involved 20 participants recruited through online LGBTQ+ communities and referrals. Most participants originated from urban areas in China, with parents who held stable employment, owned property, and possessed sufficient income to support initial transnational mobility, such as funding overseas education. Initially, potential participants were screened to ensure they voluntarily met the study’s criteria. Specifically, participants were required to self-identify as gay men and have lived in a country outside of China for at least 1 year. The study aimed to focus on Chinese gay men who had experienced the intersection of transnational mobility and family dynamics, and thus participants needed to have substantial experience with both migration and family expectations related to their sexual identity. The snowball sampling method was utilized due to the visibility and stigmatization challenges faced by the gay community in Chinese society. The recruitment process began with a few participants connected through informal networks, including online LGBTQ+ communities and personal contacts within the researcher’s network. Invitations to participate were shared within WeChat and Tencent app groups, which are widely used by the Chinese LGBTQ+ community. This allowed for a more targeted recruitment process while maintaining participant confidentiality and ensuring that diverse experiences were represented.

This study employs life story interviews (Atkinson, 1998) to collect comprehensive and sensitive personal narratives from Chinese gay men who self-identify as gay and have experiences of transnational mobility. The interviews were conducted online via WeChat or Tencent apps between March 2022 and April 2023. Interviews were conducted in a comfortable setting where participants felt at ease to share their experiences. All interviews were conducted in Mandarin Chinese to ensure participants could express themselves comfortably in their native language. Comprehensive information about the study’s purpose, data usage, guaranteed anonymity, and associated risks was provided to participants prior to the interviews, and informed consent was obtained. In order to address the core themes of the study, the interviews were guided by a semi-structured topic outline. The following are examples of the kinds of questions: In order to explore the motivation, participants were asked questions such as “To what extent is the reason for transnational mobility related to your family of origin?”. To explore how transnational mobility affected participants’ emotions and family dynamics, questions such as, “Did you notice any changes in your family dynamics after mobility?” and “How did your family’s expectations evolve over time?” were posed. Finally, to explore identity negotiation and strategies for reconciliation with family expectations, questions included, “How do you navigate the tension between your sexual identity and family expectations?” and “What strategies have you found most effective in managing family conflicts around your identity?” As a token of appreciation for their time and insights, each participant received 50 RMB. Each interview lasted approximately 1 to 1.5 h. The data collected were systematically recorded and transcribed verbatim for analysis. During transcription, all recordings were anonymized to protect participant identities. Table 1 presents basic details about the participants, including pseudonyms, age, countries, and the duration abroad.

**Table 1:** Basic background information of 20 participants.

No.	Pseudonym	Age	Countries	Duration abroad (years)
1	Bo	26	America	3 years
2	Yang	27		3 years
3	Ning	28		10 years
4	Hong	30		6 years
5	Mark	33		5 Years
6	Tao	27		2 years
7	Lun	26	Canada	3 years
8	Chang	28		6 years
9	Eddie	25		2 years
10	Tian	31	Australia	8 years
11	Hao	30		4 years
12	Fish	29		4 years
13	Song	26		1.5 years
14	Bobby	29	New Zealand	2 years
15	Shui	25	England	1 year
16	Cheng	35		2 years
17	Yun	26		4 years
18	Xing	21	Netherlands	3 years
19	Xin	27		4 years
20	Jun	22		4 years

The data were analyzed using Charmaz’s (2006) Constructivist Grounded Theory, a flexible and systematic approach that emphasizes the researcher’s active role in constructing themes through iterative coding and interpretation. This method was particularly well-suited to exploring participants’ complex experiences while maintaining sensitivity to their unique contexts. The analysis began with initial coding, where transcripts were carefully read, and codes were assigned to terms, phrases, actions, and interactions that reflected key aspects of participants’ narratives. For example, specific phrases related to “family expectations” or “mobility decisions” were coded to capture recurring patterns. Emerging themes were then identified through focused coding, where recurring patterns and key insights within individual interviews were analyzed in greater depth. To refine these themes, I employed sensitizing questions, such as, “What specific family pressures motivated participants to pursue mobility?” and “How did participants interpret mobility as resistance?” Analytical memos were written throughout this process to document reflections, explore connections between codes, and deepen insights into how participants negotiated family dynamics. Cross-case comparison followed, where transcripts were analyzed across participants to identify shared themes such as “transnational mobility as empowerment” and “family reconciliation strategies.” Axial coding was used to identify common attributes and dimensions within themes, grouping responses related to concepts like “negotiation of family expectations” and “partial reconciliation.” Finally, thematic integration was performed to explore connections between themes, such as how participants’ experiences of “rediscovering self” during mobility were linked to their later strategies for “family negotiation.” This iterative and systematic process ensured a rigorous analysis that balanced individual nuance with broader patterns. Additionally, the neo-familism framework provided a contextual lens, enabling an interpretation of participants’ strategies for navigating family dynamics within broader cultural and structural constructs. By combining grounded theory methods with a theoretical framework, the analysis effectively captured both the diversity of individual experiences and the common patterns that emerged across the dataset.

Saturation was determined using a grounded theory approach, where data collection and analysis were iterative. Initially, I planned to recruit 20 participants, in line with established norms in qualitative research (Saunders et al., 2018). Following each interview, data were immediately analyzed to generate codes and identify emerging themes. By the 15th interview, no new themes were identified, signaling that saturation had been reached. Saturation was evidenced by the consistent repetition of key themes such as family negotiation strategies and the influence of transnational mobility on reconciliation processes. These findings indicated that the data were sufficiently diverse and comprehensive to address the research questions, and that no new insights were forthcoming. It is important to note that in grounded theory, saturation is a key criterion for ensuring theoretical sufficiency. As Charmaz (2006) and Glaser and Strauss (2017) emphasize, saturation is achieved when data collection no longer yields novel theoretical insights. Therefore, I revised the original description to more accurately reflect the use of grounded theory and the process of achieving saturation as it pertains to this methodology.

As a researcher based in China with a background in sociology and a focus on LGBTQ+ studies, I acknowledge that my positionality may influence how I interpret the participants’ experiences. To address these potential biases, I engaged in a variety of reflexive practices throughout the research process. Specifically, I maintained a research journal in which I documented my thoughts, emotions, and biases during the data collection process. This allowed me to monitor how my positionality influenced my interpretation of the data. Additionally, I regularly consulted with colleagues and mentors to critically examine my assumptions and interpretations, ensuring that they remained rooted in the participants’ voices and experiences. These practices helped me maintain transparency and minimize the impact of my personal biases, ensuring that the participants’ perspectives were accurately represented in the final analysis.

## 4 Results

The results are structured around three interconnected themes, each addressing one of the central research questions. The first theme, “Conflicts emerge: intergenerational tensions in the family,” answers the first research question by examining how family tensions, such as marriage pressures and the concealment of identity, act as key drivers of transnational mobility. These tensions underscore the conflict between family expectations and individual autonomy, motivating participants to pursue mobility as a means of escape. The second theme, “‘Wandering’: transnational mobility as both an escape strategy and a self-empowerment journey,” addresses the second research question by exploring how mobility serves a dual purpose: first, as a strategy to escape family conflicts, and second, as a pathway to self-empowerment. The first sub-theme, “Transnational mobility as an escape strategy from family conflicts,” demonstrates how participants use mobility to distance themselves from restrictive family environments, while the second sub-theme, “Transnational mobility as a self-empowerment journey to increase negotiation capital with family: to reach the ‘ready’ node,” highlights how this process enables individuals to gain the confidence and resources needed to engage with their families on more equal terms. Finally, the third theme, “‘Returning’: three intergenerational reconciliation patterns of returning to the family to solve the ‘yes, but not yet’ status,” responds to the third research question by identifying three pathways of reconciliation—negotiated acceptance, partial reconciliation, and tactical withdrawal—that participants employ after returning to their families. Together, these themes trace the trajectory of transnational mobility, from its roots in family tension to its culmination in reconciliation, providing a comprehensive framework for understanding the intersection of migration, family, and identity.

### 4.1 Conflicts emerge: intergenerational tensions in the family

Engaged in a delicate intergenerational dance of concealing their gay identities, Chinese gay men find themselves ensnared in a struggle situation against societal gender norms and self-perception. Unfortunately, this struggle is often fraught with uncertainty. The unpredictable risks associated with coming out and the societal pressure to adhere to heterosexual norms through marriage upset the delicate balance of intergenerational relations, rendering gay men vulnerable to upheaval at any moment. In this tense intergenerational dynamic, mobility becomes an inevitable recourse.

#### 4.1.1 The precursor of transnational mobility: unforeseen risks in family coming out

Coming out reveals one’s gay identities, reshaping traditional Chinese family dynamics despite challenges. Tensions and conflicts between family members become nearly unavoidable. Three risks identified are impulsive disclosure, inadvertent exposure, and internalized anguish. Managing disclosures within Chinese families during coming out leads to ongoing anxiety and strain.

Impulsive self-declaration occurs mainly during the ambiguous Chinese stage of adolescence. Due to some reasons, such as the lack of marginality awareness、romantic dynamics and education pressures (mentioned by Jun, Xing, and Lun), respondents chose to reveal their sexual orientation to their families actively. The rebelliousness and expression of adolescence turned into arguments or cold violence when it encountered the traditional family orders, and Jun’s story describes a family dispute triggered by impulsiveness:

*“I came out in high school (at 16), I was arguing (with my parents), kind of because there was an education conflict. It was an opportunity for me to just come out, the result was so bad and was a big shock to my mum, she was very naturally associating gay with drug use, and I don’t even know where she got misunderstood, my parents thought I stepping into the wrong path.” (Jun 22)*

Jun’s parents’ paranoid understanding and intense reaction threw his family life into turmoil following his coming out. They demanded that he undergo discipline to alter his sexual orientation, pressuring him to conform to societal norms of being a “normal person” (Jun 22). This plunged Jun into inner turmoil as he had not anticipated the lasting identity conflict stemming from his brief act of self-disclosure. Torn between meeting his parents’ expectations and asserting his true self, Jun wrestled with this dilemma throughout his prolonged adolescence.

Accidental exposure presents a significant concern for many Chinese gay men, as it has the potential to disrupt the apparent harmony of meticulously maintained family intergenerational relations. Participants recounted several scenarios of passive disclosure, such as parents stumbling upon a stash of pornography, friends inadvertently disclosing their sexual orientation, or parents noticing subtle cues (as highlighted by participants Bobby and Tian). Song’s narrative exemplifies the inherent uncertainty associated with passive disclosure:

*“Me coming out with my sister was completely reactive. After the breakup, my ex suddenly came to my house and told my sister about my sexuality. so scary!! I was completely unprepared; my family was in an uproar, and I was depressed myself.” (Song 26)*

Accidental exposure often forces Chinese gay men to come out prematurely, resulting in an immediate consequence: a breakdown of trust within the family (Song 26). Established bonds are abruptly challenged, requiring renegotiation. Some struggle with conformity, deepening their anguish. Constantly conforming to masculine roles expected by families affects Chinese gay men like Participant Hao, stifled by suppressing their true selves. For him, pressure to come out feels like a “Stockholm sword (Hao 30),” a metaphor he uses to illustrate the double-edged nature of his predicament. On one side, Hao feels a profound obligation to maintain his family’s perceived harmony, fearing that disclosure would lead to rejection or disappointment, especially given his observations of other families’ negative reactions to their children’s sexual orientation. On the other side, suppressing his identity places immense emotional strain on him, forcing him into a continuous cycle of concealment and self-denial. Hao describes this inner conflict vividly:

*“(Tell) the family? I don’t dare. I have a lot of gay friends, and some parents take them to the doctor and always say ‘It’s all for him’, I’m always worried about my parents’ attitude and I don’t dare to say it directly.” (Hao 30)*

Coming out, therefore, signifies a reconfiguration of family dynamics and a coercive shift toward parental authority. The act of coming out often engenders a sense of defeat and reinforces heteronormative discipline, causing the gay community to lose sight of its authentic self within the family context. Rather than fostering identity construction, strained family intergenerational relations perpetuate a “catch me if you can” dynamic that reinforces concealment. The family’s role, previously characterized by instrumental support for transnational mobility (e.g., financial or logistical assistance), transforms post-coming out into a domain of propulsive and transformative pressures regarding sexual orientation. Participants often described maintaining a sense of pre-coming-out family harmony by concealing their sexual minority identities, with transnational mobility serving as a discreet strategy for navigating these dynamics. This initial concealment sometimes provided a foundation for later reconciliation processes, as participants sought to renegotiate their roles within their families.

#### 4.1.2 The catalyst for transnational mobility: divergent marriage plans

Marriage serves as a significant rite of passage within the traditional Chinese family, where parents perceive their children’s marital union as the ultimate intergenerational duty (Song et al., 2022). Consequently, they prepare financial resources for weddings and housing and often urge their children to complete this milestone. Activities like pressuring for marriage (as mentioned by Tao) and arranging blind dates are common occurrences. For Chinese gay men, the prospect of orchestrating a heterosexual marriage presents overwhelming pressure. Cheng, for instance, opts to distance himself from his family under the guise of pursuing educational advancement:

*“I’m 35 years old, almost 40 already, which is scary, especially in my hometown, you know? They used to push me to get married and stuff. I used the excuse that I had to study, and I wanted to get a higher degree. But they still whisper about what you’re going to do when you’re old. Fortunately, I’m abroad now. I don’t know what it would be like if I was staying with my parents.” (Cheng 35)*

To manage marriage pressures, gay men often resort to deception to hide their sexual orientation and ease societal expectations. For instance, Cheng attributes his reluctance to marry to his doctoral studies, prioritizing academia. Similarly, Fish enters tacit agreements with lesbian friends to meet family expectations, assuming heterosexual roles when needed. However, deceit is not a sustainable solution, and the moment of disclosure inevitably looms.

The significant deception discussed here is a “marriage of convenience” (‘*Xinghun’ in mandarin*). Gay men, lacking self-identity, often marry to address family pressures, concealing their sexuality to ease tensions and meet family expectations. For example, Fish married his lesbian friend to alleviate stress but ended up leaving the country after realizing dysfunctional family dynamics prompted him to rediscover himself. Reflecting on his experience, Fish stated:

*“After 6 months of marriage, I divorced, and my family asked me what happened. I did not want to hide anymore, so I left the country”. (Fish 29).*

Fish’s experience proves that the strategy of deceit ultimately failed to fulfill gay men’s desires for acceptance within their families and only succeeded in perpetuating their families’ adherence to normative expectations. However, the sole alternative to avoiding marriage arrangements is to disclose one’s gay identities—a daunting choice that forces gay men to engage in profound introspection of their embodied subjectivity. This process often leads to strained intergenerational relations, marked by accusations and grievances against parents. Participant Yun laments parents who blindly adhere to societal norms and cling to family ethics without considering the emotions and well-being of their children:

*“When I was in the country, I had to get married, I didn’t want to go on a blind date or ‘Xinghun’, but my parents kept arranging it anyway. Is this out of a false sense of pride? Don’t you think it’s a ‘hypocrisy in the bones’ to sacrifice your children’s happiness?” (Yun 26)*

This indictment is not uncommon, as seen in Bo’s assertion that his marital union merely serves to perpetuate the family’s hierarchical ethos.

*“(My parents) desired progeny, descendants, with scant regard for my individuality; their paramount aim was to uphold the family legacy.” (Bo 26)*

This led Bo to reflect on intergenerational affection and obligation, feeling that ‘self-authenticity’ is entangled within an intangible lattice.

The discourse on marriage contrasts with the identity crises caused by coming out, prompting Chinese gay men to awaken. More gay men realize cutting family ties, leaving hometowns, and abandoning deception are vital for self-actualization. The gravity of marriage compels these Chinese gay men to embark on a journey of self-determination.

### 4.2 “Wandering”: transnational mobility as both an escape strategy and a self-empowerment journey

In this research, I observed diverse strategies among contemporary Chinese gay men navigating conflicting sexual identities and entrenched family norms. Some adhere to parental expectations, prioritizing family harmony. Others embrace their gay identities despite parental opposition, leading to transnational mobility and family estrangement. Another approach involves temporary role-playing, balancing conformity with self-expression. Many aspire to reconcile with parents, fostering inclusive intergenerational relations. This study examines how transnational mobility empowers Chinese gay men to reconcile with their families.

Sociologists classify intergenerational relations as democratic or authoritarian (LaSala, 2002; Di Cesare and Dagkouly-Kyriakoglou, 2022). In highly authoritarian settings, Chinese gay men conform. Conversely, democratic dynamics allow identity exploration. Traditional values prompt strategies like mobility for autonomy. These ease conflicts temporarily, allowing introspection. Risks like coming out prompt reflection, and balancing family obligations. Yet, achieving equilibrium is challenging. During conflicts, gay men may choose mobility to distance themselves from parental control.

Transnational mobility, once viewed negatively, has unexpectedly revealed new opportunities. Moving to another country helps ease intergenerational conflict and ambivalence. It allows parents to provide more support, strengthening family bonds and enhancing belonging for Chinese gay men. Mobility leads to a shift from an individualistic perspective to a diverse one within the family unit, driven by education and new experiences in managing dynamics.

#### 4.2.1 Transnational mobility as an escape strategy from family conflicts

Chinese gay men of marriageable age often strive to create both physical and emotional distance from their parents (Li et al., 2010). This aligns with research showing that family dynamics strongly influence the transnational mobility of Chinese gay men (Choi, 2022). In this study, Yang and Mark’s ideas further supported Choi’s findings:

*“I went abroad, stayed away from family (in here), like a fallen leaf. They do not understand me, every day to push for marriage and emphasize that I should get married. I do not like girls, regrettably, I cannot come out of the closet because my father is more authoritarian, and he will kill me.”(Yang 27)*

*“Although my mother is willing to listen and understand me and the family atmosphere is good, I still did not come out. I worried that they would not accept, very sorry for them, but I accepted my own identity, thus I chose to go abroad, and accepting more culture and knowledge may help me understand myself better.” (Mark 33)*

Likewise, their experiences also show transnational mobility strategically aids Chinese gay men in navigating the complexities of coming out and marriage, providing a space for self-discovery amidst family expectations. For instance, Tian’s relocation clarified his sexual orientation:

*“Before moving abroad, I was unsure about my attraction; my parents pushed for a heterosexual relationship. But overseas, it became clear: I prefer men.” (Tian 31)*

Understanding sexual identities varies, prompting proactive navigation of discrepancies with prevailing gay norms.

Participants like Lun and Tian navigate complex intergenerational dynamics shaped by economic constraints, hindering open acknowledgment of their sexual orientation. The absence of stable same-sex dynamics leads to pressure for heterosexual encounters and marriages, regardless of personal inclinations. Mobility patterns shift during major educational transitions, fostering gradual financial independence. Lun elaborates:

*“Although my university is far away from home, due to its reputation, my family would like me to go. I could not receive their surveillance, and if living expenses were not enough, I could earn by myself, like doing a part-time job.” (Lun 26)*

Social structural factors, like traditional family norms, influence mobility trajectories, making transnational relocation appealing to those with substantial social capital. Chinese gay men like Bobby symbolize discontent with current contexts, actively pursuing opportunities abroad. Bobby explains:

*“I think the conservative powers in China are very rampant so that is squeezing the living space of minority groups smaller and smaller. For example, the visibility of LGBTQ+ is also getting lower on public media platforms. All LGBTQ+ university student clubs in Beijing and Shanghai are closed by the government, and some public platforms for LGBTQ+ in universities have basically disappeared, which is suffocating.” (Bobby 29)*

As China’s population ages and the demographic dividend declines, the government advocates pro-natal policies while tightening regulations affecting the LGBTQ+ community. In 2018, gay-themed content in film and TV was banned, labeling gay identities as “abnormal.” This extends to social media, targeting content deviating from the prescribed view of marriage and morality. Faced with repression, many opt for transnational mobility to cultivate their identity and pursue individualization. Hao explains:

*“Because of the domestic situation, I think in foreign countries, at least, I do not think it (sexual orientation) has any impact on me. I could enjoy three things: first, I can realize my dream life and marry the man I like; second, if I can stay here, people will not be extra discriminatory to me; third, the most important thing is that no one will bother you, talking behind your ass. Of course, families will certainly have no direct conflicts. But at home, you need to live under pressure, and always sacrifice yourself to follow your family.”(Hao 30)*

Legal protections like male–male marriage and cultural integration attract Chinese gay men to transnational mobility, offering autonomy in expressing their sexual orientation. Smith’s research on South Asian gay men emphasizes family norms, socio-cultural pressures, and religious expectations as constraints on their exploration of sexuality (Smith, 2012), this study, conducted within the South Asian Gay men diaspora in Australia, offers significant insights into how cultural and other social structural factors of influence the lived experiences of gay men from this community. Transnational mobility serves as a strategic response to mitigate tensions from family constraints, providing relief from strained intergenerational relations. Participant Ning reflects on the therapeutic value of transnational distance, experiencing family care and support:

*“After I came to America, I had more contact with my family. Sometimes I could call my mom and my grandmother several times a week and each phone call was very long. They also care about me and my partner’s condition, financial situation, and health. They also forwarded me some social issues in America and told me to be safe. I feel closer to them.” (Ning 28)*

Transnational mobility offers an escape from family conflicts, prompting a reevaluation of family values. Despite initial disillusionment, participants find belonging and contentment, transforming negativity into happiness.

Participants navigate complex tensions within intergenerational family dynamics, struggling with conceptual gaps in sexual orientation while relying on parental support. Chinese gay men reassess societal expectations, resorting to avoidance strategies. Transnational mobility emerges as a solution, driven by countries prioritizing individual freedoms. For some, relocating abroad is essential for nurturing legitimate same-sex dynamics.

#### 4.2.2 Transnational mobility as a self-empowerment journey to increase negotiation capital with family: to reach the “ready” node

Transnational mobility provides an ideal environment for strengthening the gay identities of Chinese gay men. It prompts them to recognize a crucial turning node—the ability to communicate openly with their parents. This reinforced gay identity becomes a significant asset for negotiating with older generations in the family, easing the “returning” process.

The “ready” node, drawn from participant narratives, includes financial independence, education, and stable dynamics, bolstering gay men’s identity. Many hesitated to come out before leaving home, lacking confidence and support for discussions with parents. Being ready for reconciliation is crucial.

Financial independence provides vital social capital, freeing individuals from family constraints. Lun stressed its importance, noting that a steady income grants him decision-making agency and relieves worries about relying on his parents financially. Lun articulated:

*“It’s best to raise my (sexual minority) status with them when I’m financially independent. Not fully independent yet now. I have to at least make my mom feel that I can live well in the future before I talk to them. It’s more important to get rid of it (family) to support me financially.” (Lun 26)*

Without financial independence, participants such as Lun hesitate to discuss their sexual orientation with parents, fearing potential conflicts leading to withdrawal of financial support, especially during schooling. Consequently, many pursue internships and jobs cautiously to achieve autonomy.

During adolescence, cognitive processes stabilize gradually, shaped by societal norms and self-perception in forming gay identities. This identity formation combines external social values and internal awareness. Education offers a diverse knowledge base and inclusive setting, aiding gay men in understanding their identity. Ning highlights the significance of educational attainment:

*“I’ve been in the US for 10 years and just got PR. I’ve been here for a long time, but nothing seems to have worked out, I’m not ‘ready’! My mom wanted me to have a child before I turn 30. I’m still doing my PhD degree, so I must get my education done first. And I’ve gained a lot of cultural knowledge about gay pride, my friends and colleagues at school are supportive of me showing who I am, and I’m getting better at planning my future.” (Ning 28)*

Educational attainment is crucial in accessing identity knowledge and fostering an inclusive atmosphere, significantly aiding gay men in understanding their gay identities. Chang, whose PhD project focuses on sexual minorities, highlighted another important aspect of educational achievement: it provides formal avenues for communication.

*“Because my PhD was on sexual minority health and trauma, and some domestic violence, when I started doing this, I would talk to my parents a little bit more directly. I found that they would listen to me from a more scientific perspective.” (Chang 28)*

Pursuing higher education amplifies awareness of sexual diversity, as noted by Chang, aiding young Chinese gay men in legitimizing their identity. Additionally, higher education enables smoother communication between Chinese gay men and their parents. The dissemination of scientific knowledge serves as a powerful means to challenge parents’ singular perspectives gradually, fostering essential transformations in intergenerational relations.

Additional evidence from Hong highlights the positive impact of stable partnerships on identity development. Attending an LGBTQ+ event during his graduate studies in America prompted Hong to deeply reflect on the importance of long-term same-sex dynamics within family intergenerational dynamics.

*“When I first came to America to do my Masters, I did an event to work with the LGBTQ+ community and it occurred to me that your parents don’t approve of you really because they’ve never understood this community. So, I think that when having a partner, you can show your happy life with your partner to your parents, they might understand and accept it.” (Hong 30)*

To a considerable extent, a stable partnership cultivates profound happiness on a broader scale. Intimacy within a gay partnership nurtures self-acceptance and prompts reassessment by parents. Bobby observed that, although his parents “may” have been aware of his sexual orientation, their parental authority stifled open discussion. A stable partner empowers Chinese gay men to confidently embrace their identity, fostering genuine engagement from heterosexual parents. Moreover, it exemplifies that life can be fulfilling irrespective of adherence to heterosexual norms, progressively challenging parental orthodox beliefs.

In transnational mobility, three interrelated factors converge to bolster the gay identities, guiding Chinese gay men to a pivotal “ready” node. Obtaining employment and achieving financial independence symbolizes the first stride toward “gaining bargaining power, “as illustrated in the study. This newfound autonomy liberates Chinese gay men from sole dependency, allowing them to engage with their parents on an equitable footing.

Concurrently, educational attainment provides a diverse and inclusive knowledge base, fostering awareness of diverse groups and advocating sexual equality. Furthermore, stable same-sex intimate dynamics serve as a source of happiness, challenging prevailing heteronormative standards. Together, these factors bolster and refine individual identities, leading to the achievement of the “ready” node. Throughout this journey, each gay man progressively embodies an ideal state of existence.

The concept of “Becoming” (Worth, 2009) proves invaluable in transcending the constraints of linear life-stage development, integrating geographic mobility into the framework of youth transition as an evolving and embracing experience characterized by variability and instability. Transnational mobility thus offers participants a journey of becoming, as expressed by Hao:

*“My family would urge me to have children, but I don’t think it’s time to get to that stage yet. So, I left the country, and it would be better if I could stay here and get a PR. I have now found a partner and a job, and I have told my parents.” (Hao 30)*

Transnational mobility empowers Chinese gay men, offering opportunities for identity advancement and family dialogue. Exposure to tolerant environments fosters individualization. Characters like Ning, Lun, Hong, and Hao highlight the importance of readiness before discussing sexual minority status with parents. Accumulating economic or cultural capital during mobility showcases their capacity to excel in non-normative contexts, echoing Yan’s observations on neo-familism. In contemporary Chinese society, the family structure emphasizes children, who prioritize their own interests while maintaining family connections. Worth suggests mobility facilitates self-realization, culminating in readiness for authentic family negotiations and self-acceptance (Worth, 2009).

However, even after transnational mobility, some gay men remain in the “yes, but not yet” status, as described by Luo et al. (2024), indicating that they have reconnected with their gay identities through transnational mobility. Nevertheless, they still seek to establish their position within the family after relocating across borders, just like the fallen leaves returning to their roots. In the subsequent section, I will elucidate how transnational mobility aids Chinese gay men in finding their place within the family, helping to overcome the alienating perceptions of their parents entrenched in traditional Chinese norms and fostering reconciliation within strained intergenerational relations.

### 4.3 “Returning”: three intergenerational reconciliation patterns of returning to the family to solve the “yes, but not yet” status

Sociologists emphasize the enduring significance of the family unit for individual well-being and a sense of belonging, highlighting its indispensable role (Hindman, 2019; Kong, 2020). Consequently, amidst conflicts, Chinese gay men feel compelled to seek reconciliation with their families, rooted in notions of love. Transnational mobility, originating and culminating within the family sphere, goes beyond physical return, fostering the establishment of new dynamics and self-acceptance. In this section, I outline potential return pathways Chinese gay men may pursue upon reaching the pivotal “ready” node.

Gay identities’ impact on intergenerational relations extends beyond individuals, influencing family dynamics and encountering social pressures from extended networks. Parents, constrained by societal norms of heterosexual orthodoxy, struggle to accept gay identities in a society that often rejects it. Caught in a dilemma, they cannot bear to see their children face social ostracization, their protective instincts triggered by external hostility. Acknowledging this, parents undergo a significant change in their anti-family attitudes, fostering empathy for each other’s struggles and the underlying love amid conflict. Enduring societal judgment, individuals reflect on the past, prompting a reshaping of family dynamics.

Transnational mobility prompts mutual understanding. Parents grasp children’s identities, while children empathize with parental pressures. This understanding catalyzes positive reconciliation. Three patterns emerge: negotiated, forceful, and disengaged. Negotiated reconciliation involves collaborative family adjustments. Forceful reconciliation stems from offspring pressuring parents. Disengaged reconciliation prioritizes self-growth. Ultimately, reconciliation transforms, fostering renewed family dynamics.

#### 4.3.1 Negotiated reconciliation

Reconciliation may be facilitated through negotiation, as mutual understanding fosters the dissolution of strained intergenerational relations. With a stable and fortified gay identities, individuals assertively redefine their position and role within family dynamics. Parents faced with significant global distance from their children and constrained by prevailing social norms, increasingly find negotiation challenging, as noted by Bo. Hence, negotiation emerges as an inevitable trend in the process of reconciliation:

*“When you go abroad, you can feel that geographical distance limits the expression of emotions. This is inevitable. And because my parents and I are in different societies, different cultures have different constraints and evaluations on people and sexual minorities, so I must work hard to find the possibility of communicating with them in the differences.” (Bo 26)*

Having solidified their gay identities and understanding of sexual diversity, participants strive to reconnect with their families after transnational mobility, seeking acceptance. Equipped with tools for constructive dialogue, they address intergenerational tensions, prepared to disclose their sexual minority identity with parental support. The focus lies on reconciliation, aided by gained autonomy and social resilience. They appreciate parents’ supportive roles, as articulated by Eddie:

*“I think the biggest obstacle to my reconciliation with my parents comes from the fact that they feel pressure to be a sexual minority parent, that they will not be able to be respected, some relatives will say some make them ashamed. In fact, as for me, I have already gone abroad, so those nosy ones can’t affect me, but not necessarily my parents, they will be harder.”(Eddie 25)*

The negotiated process unfolds in two stages. Initially, Chinese gay men assimilate their “gay” identity into family dynamics, disrupting entrenched norms and adjusting dynamics. Transnational mobility empowers gay men to express themselves genuinely within intergenerational contexts. Negotiating involves dialogues on companionship, fertility, and retirement, reaching a consensus on accepting same-sex partners, and navigating evolving family structures. Acknowledging challenges in parental acceptance and adherence to norms requires transnational support and financial independence. Shifting to democratic family models promotes shared values and integrates gay identities.

*“Because I really hope they can support me (on this issue), I keep hinting to them that homosexuals can also be happy. By educating them about the protection of sexual minorities in foreign countries, I think they have changed their minds. It wasn’t easy, but through repeated conversations and understanding, I finally got my family to respect my choices.” (Xin 27)*

Xin’s negotiation experience shows that the first step in reconciliation negotiations is crucial. They did not “rush” their sexual minority identity into the family directly to shock their parents, but first tried to hint at it from other aspects and let their parents slowly accept it through constant communication.

The second aspect concerns negotiating marriage expectations. Before transnational mobility, strained intergenerational relations often pressured gay men into conforming to heterosexual norms and traditional marriages. However, individuals embracing their authentic selves demonstrated to their parents, through same-sex intimacy, that happiness could be found outside heterosexual marital norms. This evidence challenges the link between marriage and happiness, emphasizing personal experience over societal conformity. Observing same-sex couples thriving without formal marriage, parents realize their children’s capacity for companionship and support. Consequently, there’s a shift in marriage expectations, focusing on nurturing children’s well-being. While love-based marriages deserve blessings, same-sex dynamics rooted in love also merit recognition as sources of genuine happiness. Bobby made his parents support “marriage” and then “love” by constantly communicating and bringing his boyfriend to the front stage at the right time.

*“I always feel that they can more or less sense some problems, and I am happy to communicate with parents about some rights issues, so in previous communications, I found the possibility of reconciliation through constant negotiation, because they sometimes show sympathy for the Chinese gay community. So, at this time I will go a step further and guide them from sympathy to support. Then I will bring up the fact that my roommate abroad is actually my boyfriend at the right time.” (Booby 29)*

The experience of Xin and Bobby illustrates these two stages very well. Therefore, negotiation disrupts established norms within intergenerational relations. Sexual identities, diverse in nature, need not conform to societal norms; marriages can embrace same-sex frameworks instead of adhering strictly to heterosexual standards. Intergenerational relations hold a central place in family values, receiving considerable attention within the family unit. Negotiated reconciliation thus signifies a transformative process as individuals strive to establish new intergenerational connections, transitioning from “you and me” to “us.” This aligns with neo-familism principles, emphasizing both individualization and intergenerational bonds.

Conflicts may arise between individualized development and maintaining intergenerational relations, posing dilemmas for families. Transnational mobility sheds light on the interplay between individuated growth and family ties. Individuals accrue identity resources through these experiences, enhancing their negotiation skills. Simultaneously, parents may shift focus from conflict to their children’s well-being amidst increased distance. This process of individuation unfolds gradually, leading to a readiness to reconnect with families with a firm gay identity, completing a journey from wandering to returning. This transition, from distancing to reconciling with family, signifies growth and maturity for both parties, affirming family bonds as havens of love and support.

#### 4.3.2 Forceful reconciliation

Reconciliation via negotiation requires heightened understanding and empathy, which can be inherently challenging. Hence, for most “ready” gay men, while emotional empathy remains vital, the redistribution of family power, alongside acquiring greater social, economic, and cultural resources, urges for change. This restructuring of intergenerational relations may lead to a new model where children assume leadership, with parents following suit.

As a government official, Shui describes the successful realization of the family landscape he envisioned:

*“When I came home from studying abroad, I came out to my family directly and told them about my marital arrangements and surrogacy plans, they didn’t dare to speak out, my hometown is conservative, and I had just become an official in XX department of the local government, and I was a part of the success of the whole family, they chose to accept it, and even helped me to withstand external pressures such as urging to get married from relatives.” (Shui 25)*

Individual success enhances family prestige, but societal status may overshadow gay identity’s significance. Shui’s story highlights neo-familism’s argument that families provide steadfast support in uncertain times, celebrating shared achievements. As offspring reshape family dynamics, parents find validation in their children’s accomplishments. There’s a noticeable trend as parents increasingly accept their children’s sexual identities, especially when gestures like Shui’s offer to surrogate a child ease parental worries and strengthen family bonds. In addition, Ning also said that because he is studying for a doctorate in the United States, his education level is higher than that of his parents, so he also has the final say when communicating with his parents at home:

*“Because my parents’ education level is not as good as mine, and my family is the type of family that values people who are good at studying, it is easy for me to take the lead in negotiations with them. I will popularize the knowledge I have learned to them, including knowledge about sexual minorities. Sometimes I don’t speak in a negotiating tone, but due to the gap in knowledge, they will listen to my leadership and then accept my identity.” (Ning 28)*

In this reconciliation pattern, transnational mobility serves as a resource arsenal, empowering Chinese gay men to establish and nurture new family ties, often leading to reduced parental authority. While Ning used the phrase “take the lead in negotiations,” his experience does not reflect a reciprocal exchange of views. Instead, his advanced educational background created a power dynamic in which he assumed an authoritative role in family discussions. The knowledge gap between him and his parents meant that his assertions about his sexual identity were met with little resistance, not because they had engaged in extensive deliberation, but because his parents largely deferred to his intellectual and social standing. Similar to Shui’s experience, Ning’s ability to assert his identity stemmed from his elevated status within the family structure rather than from a negotiated compromise. Instead of stigmatizing their children’s sexual identities, in this pattern, families celebrate their individual achievements.

#### 4.3.3 Disengaged reconciliation

Disengaged reconciliation, distinct from preceding models, reconfigures family dynamics, fostering inclusive changes and easing past traumas. Prior research shows how mobile queer individuals disengage from families to liberate themselves from trauma and oppressive intergenerational relations (LaSala, 2002). Disengagement blends temporal and spatial aspects, often as a strategic response to initial conflicts. In this study, disengaged reconciliation emerges later in mobility, following sporadic contact, as a step towards eventual reconciliation. Participants anticipate reuniting with their family.

Disengagement lays the groundwork for the reconfiguration of family intergenerational relations. As Yun pointed out, parents require time to acclimate to and accept sexual identities diverging from traditional gender norms, albeit in a rational manner.

*“When I’m able to take good care of myself and have a respectable job, I will inform my parents, and I can wait until they figure it out and come to me. We’ll then start building our beautiful family all over.” (Yun 26)*

For conservative parents, embracing new ideas and diverse inclinations is a challenge. Disengagement allows reconciliation. Unlike the initial departure driven by conflict, post-mobility disengagement is voluntary. Before leaving, Chinese gay men like Hao ensure they are prepared, acquiring scientific knowledge about gay identities and insights from countries with legalized rights. However, ongoing personal growth and maturation are still required.

*“I don’t know yet if I could take on the responsibility of re-organizing (family intergenerational relations) and (doing) things around my ideas, I approve of my sexuality, which came out to my mum and my dad has changed subconsciously, they probably know but I think I’m still young and it’s not my time to be in charge of the family, so just wait a little bit more. I think I’ll be able to deal with the family when I’m 35 years old.” (Hao 30)*

Disengaged reconciliation is not a result of emotional turmoil but intentional disengagement for reconciliation. It benefits gay men and parents, allowing them time to process new information and past conflicts.

During disengaged reconciliation, new family intergenerational relations aren’t actively reshaped. Gay men infuse the existing order with their gay identities. Participants see disengagement as an opportunity to address conflicts, gradually influencing parental acceptance. They maintain old family relations, avoiding direct repercussions of conflict management failures. *“It will be fine if the phone does not pick up for a few days.” (Song 26).*

The concept of “returning” extends beyond physical presence, symbolizing the reintegration of various sexual identities into family contexts, which is the primary source of intergenerational tensions. My focus is on how these gay identities are reintegrated and how reconciliation unfolds. Three reconciliation patterns—negotiated, forceful, and disengaged—emerge, illustrating efforts to reshape family dynamics to include gay identities. Some Chinese gay men choose to stay abroad, reintegrating their sexual identities into newly negotiated family relations, and finding their place within. Returning, marked by courage, is more appealing than narratives of family estrangement. Transnational mobility helps shed fear and stigma, fostering a balanced family dynamic. Ultimately, they resolve tensions and redefine family as unified and loving. Negotiation is central, empowering participants to navigate individual growth and conflicts, and cultivating harmonious intergenerational relations.

## 5 Discussion and conclusion

This study departs from the binary framework of acceptance and alienation that is prevalent in previous research on queer identity and family dynamics. Through a more flexible approach, this study focuses on the transnational mobility of Chinese gay men due to family and the neglected “reconciliation” in the post-transnational mobility process. Under the framework of neo-familism, this study takes an alternative approach to studying the reconciliation process of queer identity and family relations, describing it as a range that includes partial compromise, forceful disclosure, and disengagement, making significant contributions to the fields of queer immigration studies and family sociology. These findings highlight the various ways in which transnational mobility serves as a transformative force in family systems, reshaping expectations and facilitating opportunities for intergenerational dialogue. While previous studies have focused primarily on individual identity formation in transnational contexts (Choi, 2022; Kong, 2019; Yu and Blain, 2019), this study shifts the focus to the interaction between mobility, cultural expectations, and family negotiation.

Drawing upon research in queer studies and gender identity negotiation among transnationals, this study further contributes to globalizing queer migration research. The findings resonate with studies like (Luo, 2022; Luo et al., 2024) exploratory research on the significance of *Jia* for marginalized groups in China through the framework of neo-familism, which demonstrates how mobility helps Chinese gay men challenge entrenched family norms. In particular, this study extends neo-familism by showcasing how Chinese gay men use mobility to navigate their sexual identities while reconciling with family expectations. Additionally, similar to the negotiation of femininity among Indian and Pakistani immigrant women (Chaudhary, 2024), the experiences of these men reveal both shared and distinct patterns in how gender and sexuality are negotiated across borders and cultural expectations.

The three distinct patterns of reconciliation—negotiated, forceful, and disengaged—demonstrate that reconciliation is not a uniform or linear process. These findings indicate the complexities of negotiating queer identities within family dynamics, where transnational mobility can either facilitate acceptance or reinforce traditional roles. This study expands on the dominance of Western-centric perspectives in queer migration literature, offering new insights into the specific strategies Chinese gay men employ to navigate family dynamics in a non-Western context.

Nevertheless, several limitations must be considered. The sample of 20 participants from urban areas is not fully representative of the broader Chinese gay male population, and the reliance on self-reported data introduces biases that must be acknowledged. Future research could address these limitations by including a broader, more diverse sample, exploring how rural or non-migrant individuals navigate these processes. Furthermore, the complexities of reconciliation should be explored beyond the three categories identified in this study. While the three reconciliation patterns (negotiated reconciliation, forceful reconciliation, and disengaged reconciliation) are presented as distinct for the sake of analysis, they should not be understood as mutually exclusive or fixed categories, because these three reconciliation patterns may not fully capture the complexity of the real-world experiences of the participants. Rather, they represent ideal types that were dominant at certain points in participants’ lives, and participants may shift between strategies based on changing social, family, or personal contexts. The experiences of participants are likely not static; as their life circumstances evolve, so too may their approaches to negotiating family dynamics. This variability is a central aspect that could be explored in future research.

In conclusion, the findings underscore the transformative role of transnational mobility in renegotiating family ties for Chinese gay men. The resources acquired through mobility—such as economic independence and education—empower participants to challenge traditional family structures and engage in dialogue. As such, this study contributes to our understanding of queer migration and family sociology, emphasizing the need for future research to consider more diverse transnational and cultural contexts in exploring identity negotiations.

## Data Availability

The raw data supporting the conclusions of this article will be made available by the authors, without undue reservation.
